# Dry Eye Disease: Emerging Approaches to Disease Analysis and Therapy

**DOI:** 10.3390/jcm8091439

**Published:** 2019-09-11

**Authors:** Mostafa Heidari, Farsad Noorizadeh, Kevin Wu, Takenori Inomata, Alireza Mashaghi

**Affiliations:** 1 Basir Eye Health Research Center, Tehran 1418643561, Iran; mostafahaidari70@yahoo.com (M.H.); farsadnoorizadeh@gmail.com (F.N.); 2 Farabi Eye Hospital, Department of Ophthalmology and Eye Research Center, Tehran University of Medical Sciences, Tehran 133661635, Iran; 3 Department of Ophthalmology, Icahn School of Medicine at Mount Sinai, Ophthalmic Consultation Service, New York, NY 10029, USA; 4 New York Eye and Ear Infirmary of Mount Sinai, New York, NY 10003, USA; 5 Department of Ophthalmology, Juntendo University Faculty of Medicine, Tokyo 1130033, Japan; 6 Department of Strategic Operating Room Management and Improvement, Juntendo University Faculty of Medicine, Tokyo 1130033, Japan; 7 Systems Biomedicine and Pharmacology Division, Leiden Academic Centre for Drug Research, Leiden University, 2333CC Leiden, The Netherlands; 8 Department of Chemistry and Chemical Biology, Harvard University, Cambridge, MA 02138, USA; 9 Department of Ophthalmology, Shanghai Medical College, Fudan University, Shanghai 200000, China

**Keywords:** dry eye disease, immunometabolism, microbiota, omics, eye-on-a-chip, clinical signs, DED treatment

## Abstract

Dry eye disease (DED) is among the most common ocular disorders affecting tens of millions of individuals worldwide; however, the condition remains incompletely understood and treated. Valuable insights have emerged from multidisciplinary approaches, including immunometabolic analyses, microbiome analyses, and bioengineering. Furthermore, we have seen new developments in clinical assessment approaches and treatment strategies in the recent past. Here, we review the emerging frontiers in the pathobiology and clinical management of DED.

## 1. Introduction

Dry eye disease is a multifactorial disease of the ocular surface [1]. The disease imposes a major healthcare burden, given its high prevalence [2,3] with tens of millions of individuals seeking eye care [4]. This condition is characterized by a dry, gritty, or burning feeling in the eye, as well as excessive tearing and photosensitivity [5], thereby compromising clear vision and decreasing quality of life. Several factors including dryness, trauma, air pollution, and smoke, allergens, dysbiosis, and UV light, can increase tear osmolarity and destabilize the tear film, resulting in sensory stimulation and irritation of the ocular surface [6]. This process triggers inflammatory pathways, recruits immune cells, and leads to cytokine overexpression at the ocular surface, resulting in a vicious cycle that further damages the area. The Tear Film & Ocular Surface Society International Dry Eye Workshop II (TFOS DEWS II) released a report in 2017 that classified dry eye disease (DED) into two major classes: aqueous tear-deficient DED and evaporative DED. The former can itself be classified into two subclasses as Sjögren syndrome (SS): DED and non-SS DED [7].

Despite progress, DED remains incompletely understood and treated. Interdisciplinary approaches have been increasingly applied to resolve DED complexity and to develop new diagnostic and treatment strategies. Novel concepts and tools from analytical chemistry, engineering, immunology, and microbiology are being introduced and examined for the treatment of DED. Here, we review the developments in DED treatment and care in the last five years, with particular emphasis on emerging concepts and interdisciplinary approaches that may significantly impact the field in the future.

## 2. Emerging Insights into DED Pathogenesis

In recent years, we have witnessed the emergence of a new research frontier at the interface of immunology and metabolic studies. The field of “immunometabolism” has already produced a substantial number of discoveries and contributed to disease analysis [8]. Immunometabolic analyses have in particular generated insights into autoimmunity including rheumatoid arthritis [9] and Lupus erythematosus [10]. What is emerging is a delicate interplay between metabolic reprogramming and immune signaling, which is providing an extra dimension to our understanding of the inflammatory processes [11,12].

The field of immunometabolism is new to ophthalmology. There has been a surge in this type of research in other areas of medicine, including neurology (e.g., Parkinson’s), cardiology (e.g., atherosclerosis), and geriatrics [13]. A similar approach could be highly valuable for ophthalmology and in particular, for studying the inflammatory eye processes. However, this notion is yet new to the field, and most researchers in ophthalmology have limited knowledge of this emerging area.

DED is an inflammatory disease that involves both metabolic and immune dysregulation. The ocular surface is home to different microbial organisms that contribute to the metabolism of the ocular surface. Pathological alteration of the microbial composition of the ocular surface can induce an immune response. In the following sections, we discuss the changes in the metabolism, immunity, and microbial composition associated with the pathogenesis of DED. This review strives to address the gap in the understanding of the pathogenesis of DED and to identify the opportunities for improving the treatment and care of DED.

### 2.1. DED-Associated Metabolic Dysregulation

**Figure 1:**
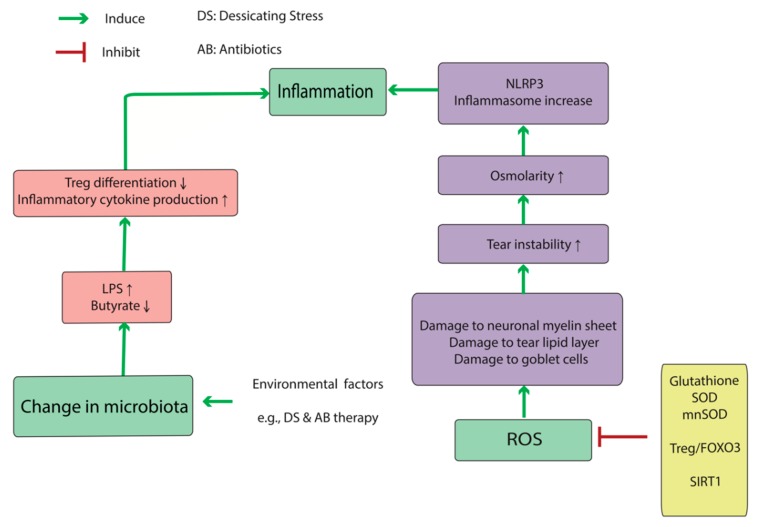
Oxidative metabolism and changes in normal microbiota contribute to dry eye disease (DED) by inducing inflammation at the ocular surface. Reactive oxygen species (ROS) directly or indirectly activates the NLRP3 inflammasome by increasing tear-film instability and osmolarity. DED-associated changes in the microbiota is in turn associated with changes in the metabolic profile of the ocular surface which changes the balance between pro- and anti-inflammatory arms of the immune system toward the proinflammatory pathways. The induced inflammation is presumably the cornerstone of DED pathology. Abbreviations: DED: dry eye disease, FoxO3: Forkhead box O3, LPS: lipopolysaccharide, MnSOD: manganese superoxide dismutase, NLRP3: NLR family pyrin-domain-containing 3, ROS: reactive oxygen species, Sirt1: sirtuin 1, SOD: superoxide dismutase, Treg: regulatory T-cell.

Oxidative stress activates the pathologic cascade associated with DED at several points (Figure 1). Reactive oxygen species (ROS) damages goblet cells, myelin sheaths of ocular-surface nerves, and the tear lipid layer, which in turn results in tear-lipid instability and inflammatory dysregulation [14]. Tear instability causes increasing osmolarity, which can induce oxidative conditions at the ocular surface. When primary human corneal epithelial cells (HCECs) were exposed to hyperosmolar media, reactive oxidative substances increased significantly, as measured by 2′,7′-dichlorofluoresceindiacetate, a membrane-permeable substance that can be oxidized in cells during oxidative stress to produce highly fluorescent 2′,7′-dichlorofluorescein (DCF). 4-Hydroxynonenal (4-HNE) and malondialdehyde (MDA), the toxic products of lipid peroxidation, increased in a dose-dependent manner as the media osmolarity rose, and there were decreases in anti-oxidative enzymes, such as SOD and glutathione peroxidase (GPx), in HCECs after exposure to hyperosmotic media [15]. Upon HCEC culturing in hyperosmotic media (550–550 mOsm), another study showed that DCF staining and NLR family pyrin-domain-containing (NLRP)3 inflammasome mRNAs, including *NLRP3*, apoptosis-associated speck-like protein (ASC), pro-*caspase*, and pro-*interleukin* (*IL*)-*1β*, increased. Furthermore, these elevations were inhibited by adding *n*-acetyl cysteine as an antioxidant to the medium. Interestingly, silencing *NLRP3* expression via small-interfering RNA in HCECs exposed to a hyperosmotic state attenuated levels of *ASC*, pro-*caspase*, and *IL-1β* mRNA levels. All of these components, as well as ROS levels, are elevated in DED patients [16]. Chi et al. [17] reported increases in *IL-1β* and *IL-18* expression after reduction of NLRP3 levels by glybenclamide treatment in mice exposed to desiccating stress (DS). Additionally, treatment with a caspase-1 inhibitor (z-YVAD fmk) did not alter NLPR3 activity but significantly restored NRLP6 production (an inflammasome component) downregulated by hyperosmolar stress. Moreover, caspase-8 can stimulate NLRP3 activation, as well as its downstream pathways. These findings showed that hyperosmolar stress accelerates an immune cascade via oxidative stress, thereby offering several targets for potential prevention and treatment of DED.

There are several other anti-oxidative mechanisms that compensate for oxidative stress. Liu et al. [18] measured the levels of sirtuin 1, FOXO3, and manganese superoxide dismutase (SOD) proteins in diabetic mice with DED, observing elevated levels at weeks 1 and 4 in the diabetic DED group as compared with those in the non-DED group, although the levels decreased by week 8. They concluded that this change was due to compensatory mechanisms to increase the levels of anti-oxidative molecules in diabetic mice, but by week 8, the anti-oxidative system was exhausted.

### 2.2. DED Immunity and Immunometabolism

According to the current models, T helper (Th)17 (IL-17-secreting CD4+ T cells) and Th1 cells are the major immune mediators of DED [19] and are recruited to the ocular surface by C-C motif chemokine receptor (CCR)6 and CCR3, respectively. Coursy et al. [20] reported that DS increases the population of CCR6+CD4+ and CCR3+CD4+ T cells at the ocular surface and in regional lymph nodes, resulting in their respective secretion of IL-17 and IFN-γ. Moreover, ablation of these two receptors avoids corneal barrier disruption, T-cell infiltration, and GC loss in response to DS. Additionally, CCR3 ablation avoids corneal barrier disruption and T-cell infiltration but does not decrease the GC loss in response to DS, whereas IFN-γ secretion does decrease GCs in DED [21,22].

Th17 cells migrate to the ocular surface by expressing CCR6 on their surface. CCR6 binds to C-C motif chemokine ligand (CCL)20, which is expressed on the ocular surface epithelium and upregulated in DED patients. However, blockade using an anti-CCL20 antibody decreases Th17 propagation and infiltration of the ocular surface in DED and improves clinical signs of DED while decreasing the cytokine expression (IL-6, IL-23, TNFα, and IFN-γ). CD11b+ cells are antigen-presenting cells (APCs) recruited to the cornea during inflammatory conditions. When treated with subconjunctival injection of an anti-CCL20 antibody, CD11b+ cell infiltration to the cornea decreases [23]. Additionally, treatment with an antibody against granulocyte-colony-stimulating factor reduces in vivo and in vitro migration and maturation [expressing major histocompatibility complex (MHC) II] of CD11b+ dendritic cells (DCs) at the ocular surface and improves clinical signs of DED in murine models. Furthermore, granulocyte-macrophage colony-stimulating factor (GM-CSF) recruits CD11b+ APCs to the ocular surface. It has been shown that Th17 cells are the sources of upregulated GM-CSF in DED at the ocular surface [24].

IFN-γ is an inflammatory cytokine secreted by the Th1 lymphocytes and mediates cellular changes during DED progression [25]. IFN-γ increases aqueous tear deficiency (ATD; both Sjögren and non-Sjögren), with the IL13:IFN-γ ratio decreasing in both ATD groups as compared with that in controls [26]. Mucin proteins promote GC density, and *mucin 5AC* (*MUC5AC*) transcripts were lower in both ATD groups while levels of conjunctival IFN-γ were negatively correlated with tear meniscus (TM) height (TMH) and conjunctival GC density. Additionally, small proline-rich protein 2G transcripts were higher in patients with ATD and positively correlated with IFN-γ levels. IFN-γ and IL-13, the inflammatory cytokine release by Th2 lymphocytes, stimulates proliferation of less-differentiated GCs and the expression of *MUC5AC* [27]. In addition, IL-13 stimulates the expression of the Fas ligand, CCL26, chloride channel calcium-activated 3, trefoil factor 3, and restin-like molecule β, which function as an apoptotic receptor on lymphocytes, a chemotactic factor of CD4+ Th cells, eosinophils, and basophils, a marker of GC hyperplasia and secretory activity, and a repair and maintenance factor for mucosal epithelial-barrier function, respectively. IFN-γ inhibits the GC proliferation in murine models [22] and increases levels of proteins involved in the unfolded protein response (UPR), which inhibits *MUC2* and *MUC5AC* mRNA translation. Dexamethasone treatment of GC cultures reduces IFN-γ-mediated caspase-3 and UPR-related activities, thereby preventing attenuated MUC5AC levels. Glucose-regulated protein 78 kD and spliced X-box-binding protein-1 are significantly increased in the conjunctival epithelium of patients with SS. Garcia-Posadas et al. [28] found that IFN-γ increases the intracellular calcium concentrations but inhibits cholinergic intracellular increases of calcium. Additionally, long-term elevations in IFN-γ levels prevents cholinergic stimulation of GC-related mucin production by activation of the Janus kinase (JAK)-signal transducer and activator of transcription (STAT) signaling pathway.

Chen et al. [19] reported that conjunctival mRNA expression of Th17-associated cytokines (*IL-17A*, *IL-6*, and *IL-23*) were elevated in DED patients, with *IL-17A* and *IL-6* higher in Sjögren DED than in non-Sjögren DED and no significant difference in *IL-23* levels between groups. Interestingly, the levels of these cytokines correlated with DED-specific ocular-surface parameters, such as ocular surface disease index (OSDI), tear-film break-up time (TBUT), the Schirmer I test, and cornea fluorescein staining [29].

IL-17 secreted by Th17 cells triggers the B cell proliferation and differentiation, and both Th17 and Th1 cells significantly increase B cell proliferation; however, Th17 is more functional than Th1 in this regard. IL-17 increases the proliferation of autoimmune B cells after stimulation with anti-CD40 and anti-IgM antibodies, which also increase the expression of the IL-17 receptor on the B cell surface. Additionally, IL-17 and IFN-γ secretion increase the B cell differentiation and proliferation as dominant factors in promoting DS-induced ocular-surface damage [30].

Although the adaptive immune response promotes DED-specific inflammatory processes, primary activation of this cascade does not require Th cells. DS induces MHC I-related protein A and protein B production, which activates the natural killer (NK) cells that release IFN-γ, thereby triggering epithelial cells of the corneal conjunctiva to produce Th1-related chemokines to promote the infiltration of Th1. However, the absence of Th1 cells does not inhibit the initiating phase of this process, indicating that the innate immune system is critical in initiating IFN-γ release to produce Th1-related chemokines [31].

Both CD4+ and CD8+ Tregs suppress the inflammatory process in DED [32]. In murine models, depletion or inactivation of CD8+ T cells by anti-CD8 antibodies promoted corneal infiltration of CD4+ T cells and their increased levels in draining cervical lymph nodes (CLNs). Furthermore, IL-17A production by both ocular-surface cells and CD4+ T cells increased, whereas IFN-γ production decreased in ocular-surface cells and IL-13 levels decreased in CD4+ T cells. Additionally, CD8+ T-cell depletion increases Th17 cell pathogenicity and increases IL-17 and CCL20 production and subsequent matrix metalloproteinase (MMP)-3 and MMP-9 levels, which play a central role in DS-induced corneal barrier disruption.

Under DS conditions, levels of CD8+CD103+ T cells increase and play an immunoregulatory role at the ocular surface and in CLNs; however, co-transfer of CD8+CD103+ Tregs does not suppress Th17 pathogenic response at the ocular surface, indicating that these Tregs suppress the production of pathogenic Th17 cells before their initiation by suppressing DC activation [31].

**Figure 2:**
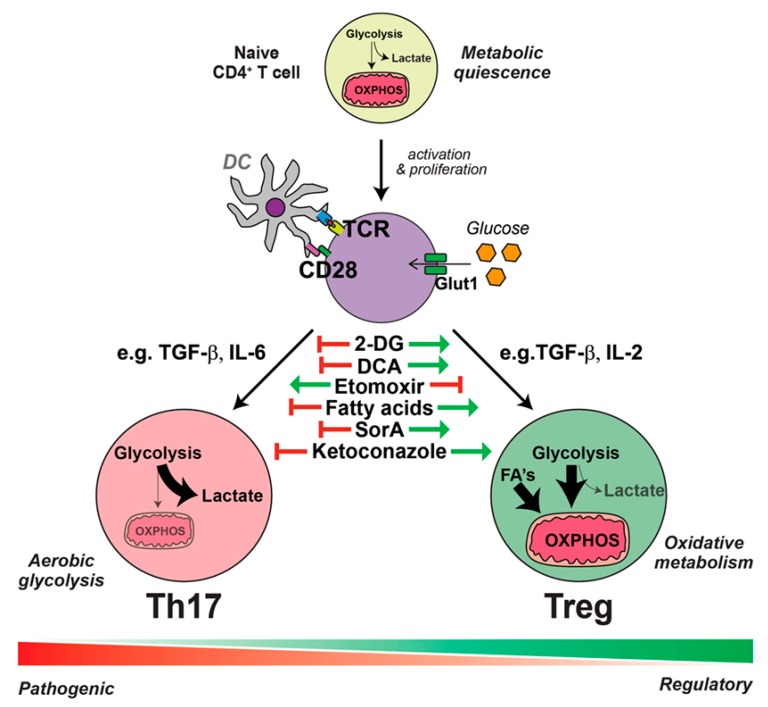
Metabolic requirements of Th17 and Treg responses. Th17 cells are dependent on aerobic glycolytic metabolism. Inducers of lipid oxidative metabolism inhibit Th17 cell generation. Conversely, Treg generation is enhanced by treatments that promote lipid oxidative metabolism and suppressed by inhibitors of lipid transport such as etomoxir. Cholesterol derivatives are required for Th17 cell differentiation and blockade of cholesterol biosynthesis, for example, with ketoconazole, suppresses the generation of Th17 cells but has no effect on Tregs. Figure taken from Binger, K.J. et al. with permission [33].

Metabolic components of the microenvironment markedly contribute to the differentiation of CD4+ T cells into Th17 or Treg cells (See Figure 2). Under Th17-promoting conditions, CD4+ T cells undergo glucose uptake and a shift to aerobic glycolysis; however, in the presence of anti-inflammatory conditions, CD4+ T cells take-up lipids and oxidize lipids for energy production [33]. The inflammatory microenvironment which results from DS conditions can transform Tregs to “exTreg” cells. The phenotypic plasticity of CD4+CD25+forkhead box P3 (Foxp3)+ T cells makes them unable to express Foxp3 in an inflammatory microenvironment [34]. These exTregs express IL17 and IFN-γ and do not suppress the inflammatory response [35].

Another principal part of the innate immune system is the promotion of inflammation in DED via the neutrophil extracellular trap (NET), which is a complex comprising extracellular DNA (eDNA), histones, cathelicidin, and neutrophil elastase. eDNA is released from dead epithelial cells, which increases in DED because of increased epithelial-cell turnover, and as neutrophils are an essential source of NET formation. Moreover, nuclease and DNase I activity is decreased in the tear films of DED patients, which also decreases the eDNA degradation. These data suggest that NET formation and accumulation in tear films are a primary source of ocular-surface inflammation in DED [36].

Currently, LFA-1/ICAM-1 interaction is being developed as a new therapeutic target in DED [37]. The LFA-1/ICAM-1 interaction plays a vital role in the cell-mediated immune response and inflammation associated in the immunoinflammatory pathway of DED. Blocking the binding of LFA-1 and ICAM-1 with Lifitegrast could be a novel approach to targeting ocular surface cell-mediated immune response and inflammation.

### 2.3. DED-Associated Changes in Normal Microbiota

**Figure 3:**
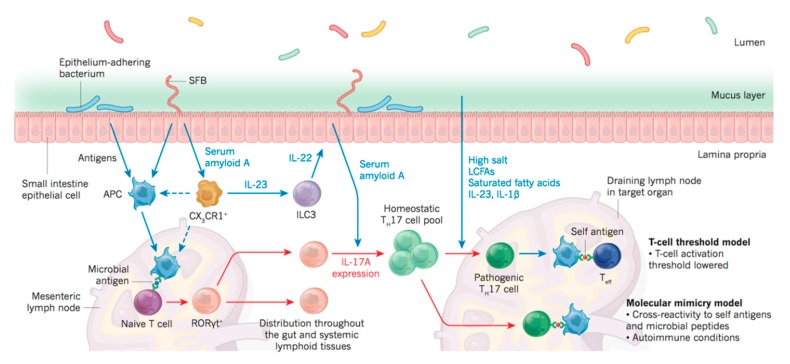
Microbiome and Th17 autoimmunity. Research in intestinal mucosal microbiome has revealed a link with Th17 response and epithelial integrity. These studies may provide useful hints to researchers working on other mucosal linings. Furthermore, direct links between the intestinal microbiota and ocular diseases have been suggested, including a link with uveitis [50], and dry eye disease [49]. Figure taken from Kenya Honda et al. [51] with permission.

An important emerging frontier in studying mucosal inflammation is the microbiota analysis. Human body harbors a significantly diverse microbiome of at least 1000 species [38]. 10–100 trillion microbial cells are distributed in skin, and mucosa of ocular, nasal, oral, and reproductive organs and co-exist in a delicate balance with the host immune system [39,40]. Direct crosstalk between resident microbes and host immune cells in mucosa emerges as a key determinant of inflammatory responses in disease conditions [41]. Th-17 and T-reg cells are in particular affected by the human microbiota (see Figure 1 and Figure 3). Furthermore, the microbiota is a major contributor to the muscosal metabolism. Resident bacteria generate a wide range of metabolites and participate in drug metabolism at the mucosa, thus affecting immunometabolic processes and pharmacotherapy [42,43,44,45].

There are several idiopathic diseases involving the ocular surface, including but not limited to DED, follicular conjunctivitis, pterygium, and Thygeson’s disease. Inflammatory dysregulation is a basic element of all such diseases, and it is logical to assume that dysregulation of the normal ocular microbiome contributes to many or all of these conditions [46]. Changes in the mucosal microbiome alter the mucosal immunity and host response to environmental insult, thereby initiating an autoimmune response, such as inflammatory bowel disease [47,48]. Paiva et al. [49] reported a decrease in operational taxonomic units in the stool of mice receiving antibiotics (AB) and exposed to DS after 10 days, with decreases in Bacteroidetes and Firmicutes phyla and increases in the Proteobacteria after DS. Additionally, they reported significant decreases in Blautia, Alistipes, Lactobacillus, Allobaculum, Bacteroides, Desulfovibrio, Intestinimonas, and Clostridium as compared with significant increases in Enterobacter, Parasutterella, Escherichia/Shigella, Pseudomonas, and Staphylococcus. Moreover, mice receiving AB + DS displayed increased goblet cell (GC) loss, infiltrating CD4+ T cells in the conjunctival epithelium, and corneal barrier disruption relative to mice subjected to DS alone. Gene expression analysis showed that AB treatment in non-stressed (NS) B6 mice increased *IL-17* and decreased *interferon* (*IFN*)-γ mRNA levels in the conjunctiva tissue relative to those in NS mice without AB treatment; however, AB + DS increased *IFN*-γ mRNA and significantly decreased *IL-13* and the *IL-13*:*IFN-γ* ratio [49]. These findings suggest that AB therapy might alter immune activity and ocular response to DS.

The ocular microbiota significantly affects the metabolic profile of the ocular surface, which in turn affects the ocular surface immunity. Short fatty acids (FAs), such as butyrate from butyrate-producing bacteria (e.g., Faecalibacteriums) play an important role in differentiating regulatory T-cell (Treg) lymphocytes [47], and increased populations of Gram-negative bacteria might account for a more severe inflammatory response in DED patients. Disruption of the ocular-surface barrier by diseases, such as DED, activate the Toll-like receptor (TLR)4 pathway [52]. Lipopolysaccharide (LPS) is an endotoxin excreted by Gram-negative bacteria and increases the expression of inflammatory cytokines in the cornea (IL-1β and C-X-C motif chemokine ligand (CXCL)10) and conjunctiva (IL-1β, CXCL10, IL-6, tumor necrosis factor (TNF)α, IL-12α, and IFN-γ) by activating the TLR4 pathway. These findings show that altered mucosal microbial diversity and mucosal dysbiosis can impact Treg differentiation [53], and production of microbial flora, such as LPS, can increase cytokine secretion from immune cells. Therefore, the data suggest that changes in normal microbiota result in an abnormal immune response (particularly via immunometabolic mechanisms), which is part of the underlying pathophysiology of DED (Figure 1 and Figure 3).

## 3. Emerging Measurement Techniques and Disease Models

### 3.1. Clinical Approaches

Despite the high prevalence of DED, there is no gold standard diagnostic approach to diagnose DED. Routine clinical exams poorly correlate with patient symptoms and are subject to observer bias [54,55,56]. Several assessments exist to evaluate the quality and quantity of ocular-surface facets and tear-unit functions; however, the set of assessments capable of diagnosing DED with acceptable specificity and sensitivity remains unknown [57]. In this section, we focus on the assessment of tear volume and tear osmolarity, which, in DED, cause oxidative stress at the ocular surface as discussed earlier. Furthermore, other recently developed techniques in DED assessment will also be discussed.

TM is conventionally assessed by the use of fluorescein staining, with Keratograph 4 capable of measuring TBUT non-invasively. Based on the interclass values and 95% confidence intervals, the between-visit and after-visit agreement of non-invasive keratography TBUT and fluorescein, TBUT does not differ significantly [58]. TMH measured from images of a fourth-generation OCULUS keratograph correlates significantly and positively with TBUT and the Schirmer test [59]. Recently, different optical coherence tomography (OCT) systems were studied to measure TMH, and Raj et al. [60] reported no significant correlation between TM area measured by Fourier domain OCT (FD-OCT), TBUT, and the Schirmer test. Additionally, Fukuda et al. [61] reported a significant correlation between upper TM volume, lower TM volume, and lower TMH with the Schirmer test but not with TBUT. Another study revealed a significant correlation between TMH measured with keratography and FD-OCT, although keratography tends to report lower results in elevated TMH [62]. Assessment of hyperosmolarity is the aim of some other measurement techniques [1,63]. Traditionally, tear-fluid osmolarity is measured by freezing-point depression and vapor pressure; however, these techniques are not visible and are limited by reflex tearing during sampling [64]. Rocha et al. [65] compared the accuracy and precision of the Wescor 5520 Vapor Pressure Osmometer, the TearLab Osmolarity System, and i-Pen for evaluating tear osmolarity, revealing that the two former devices correlated significantly with each other and were accurate and precise, whereas results from the third device did not significantly correlate with the other devices and were less accurate. Another study compared the precision and accuracy of the TearLab Osmometer using the freezing-point depression method, concluding that the results were accurate and precise at assessing osmolarity, even for hyperosmotic solutions [66]. Badugua et al. [67] introduced a novel technique to determine the individual ion concentrations in tears using silicon hydrogel, claiming that this approach can measure six dominant ionic species in the tear.

Recently, maximum blinking interval (MBI), the number of seconds eyes can stay open without blinking, was measured as an indicator of tear instability [68]. The authors reported significantly shortened MBI in the DED group as compared with the non-DED group, as well as a positive correlation between MBI and TBUT and a negative correlation between MBI and corneal fluorescein staining. They observed a sensitivity of 82.5% and specificity of 51.0% for diagnosing DED with MBI; however, further assessments are needed to confirm these results. The inter-blinking interval (IBI) is a similar evaluation that assesses the routine blinking rate of patients and is thought to be less related to corneal and conjunctival factors than MBI [68,69]. Results from IBI studies are preliminary; therefore, no conclusions have been made.

As mentioned in Section 2.1, DED-associated oxidative stress can result in tear lipid instability; thus, characterization of the Meibomian gland can be used for DED diagnosis. Keratography and OCT systems utilize infrared radiation to assess Meibomian gland loss. Using OCT, Palamar et al. [70] reported significant Meibomian gland loss in the lower eyelids of individuals with ocular rosacea rather than in healthy controls. Finis et al. [71] carried out Keratograph 5M meibography and reported that the degree of Meibomian gland atrophy in the lower and upper eyelids (Meiboscore) was significantly and inversely correlated with TBUT and positively correlated with age. Confocal microscopic imaging of the orifice of the Meibomian gland helps assess the lipid-layer thickness, using Tearscope and a Korb gland evaluator, thus serving as an alternative to evaluating Meibomian gland function [72,73]. MMP-9 is another quantifiable target for diagnosing DED [74,75]. InflammaDry is a point-of-care MMP-9 immunoassay device to qualitatively assess MMP-9 levels in tears [76,77]. One study reported a sensitivity of 85% and specificity of 94% for InflammaDry in diagnosing DED [78]. Finally, conjunctival-impression cytology (CIC) of conjunctival epithelial cells facilitates transcriptome analysis using Eyeprim, a new CIC device designed to decrease patient discomfort and anesthetic use; however, the efficacy of this device remains unknown [68,69,79]. DryEyeRhythm is a smartphone application that gathers large-scale individual real-world data and reveals risk factors including female sex, collagen disease, hay fever, depression, current contact lens use, extended screen time, and smoking, all of which contribute to the severe DED-type symptoms [80]. Internet of Medical Things (IoMT) devices such as smartphones could be implemented for telemedicine and remote monitoring approaches [81].

### 3.2. Molecular Profiling and Omics Approaches

Omics approaches (genomics, transcriptomics, and proteomics) have improved the understanding of the molecular pathogenesis of ocular diseases by providing a non-invasive, easily accessible, and individualized approach to identify the disease markers for diagnosis and treatment.

Lipidomics analyses have provided valuable information regarding DED development. Altered lipid profiles of the ocular surface are associated with clinical signs and symptoms of DED. Lam SM et al. [82] observed that normalized levels of cholesteryl sulfates (CSs), glucosylceramides (GluCers), NeuAcα2-3Galβ1-4Glcβ-Cers (GM3s), lyso-phosphatidylcholines (LPCs), and low-molecular-mass wax esters (WEs) were positively correlated with tear volume, and that absolute concentrations of these molecules decrease as tear secretion decreases, while the total concentration of tear lipids and molar fractions of phosphatidic acids (PAs) and phosphatidylglycerols (PGs) were negatively correlated with tear volume. WEs containing saturated FAs, PAs, and phosphatidylglycerols were significantly reduced in contrast to their increasing levels according to the Schirmer 1 test. This may have occurred owing to the emergence of the aforementioned lipids from the lacrimal gland. Lam et al. [83] did not observe significant differences in meibum lipids obtained from DED eyes and healthy eyes; however, the amount of unsaturated triacylglycerols, some phosphatidylcholines, glucosyceramides, and sphingolipid species were elevated in DED patients. Another study showed that 12 weeks of eyelid-warming resulted in a dramatic change in tear-lipid composition rather than the quantity of tear lipids. Lysophospholipid classes (i.e., lyso-plasmalogen phosphatidylethanolamines, LPCs, and lysophosphatidylinositols) were reduced after treatment, whereas their respective diacyl counterparts increased. Moreover, polyunsaturated FAs (PUFAs) containing diacylglycerol were reduced after treatment. Furthermore, reductions in such lipids and increases of *O*-acyl-ω-hydroxy FAs (amphiphilic lipids) correlated with decreases in evaporation rate of the cornea and sclera [84]. Another study showed that 4-HNE and MDA, two lipid peroxidation markers, negatively correlated with TBUT, the Schirmer test, tear-clearance rate, and GC density and positively correlated with kerato-epitheliopathy and symptom persistence [85].

Proteomic analysis of tear fluid is a personalized approach increasingly used to diagnose and treat DED [86,87]. Although Schirmer’s strip is the most common instrument for tear collection and protein extraction [88], it can result in sample loss. Single-unit filter-aided methods have been introduced to decrease sample loss and increase the number of proteins identified from tears [89]. Global and targeted metabolomics analyses performed on human conjunctival epithelial cells incubated in serum-free media at 280 mOsm (control), 380 mOsm, and 480 mOsm for 24 h showed that carnitine is the preferred anti-inflammatory or anti-apoptotic agent [90] while glycerophosphocholine, thought to be an osmoprotectant, is the preferred endogenous osmolyte. The results showed that increases in intermediate filament-like keratin and vimentin proteins suggested that cytoskeleton remodeling is activated under hyperosmotic stress. Additionally, they reported upregulated [heat shock protein 70-kDa (HSP70)-5, dual-specificity mitogen-activated protein kinase 3, prostaglandin G/H synthase 2, uridine diphosphate (UDP)-N-acetylglucosamine pyrophosphorylase 1, and UDP-N-acetylglucosamine, intercellular adhesion molecule 1, IL-10, IL-17, prostaglandin 2 and E2, and prostacyclins] and downregulated (plastin-2, 26S proteasome non-ATPase regulatory subunit 1, and protein glutamine gamma-glutamyl-transferase 2) proteins [90]. Another study compared the proteomic profiles of reflex tears and basal tears, observing that highly acidic proline-rich protein (PRR)4 was more abundant in reflex tears, indicating a possible protective function of this protein. These findings further the current knowledge of the metabolic markers of DED and potentially provide therapeutic targets. Another report indicated that proline-rich protein 4 and zymogen granule protein 16 homolog B protein were upregulated in reflex tears and might play a secretory role. Additionally, increased serum leakage during reflex tearing causes increased serum albumin in tears, and upregulated levels of mesothelin in tear reflex might heighten the function of MUC16, which encodes mucin. The amount of polymeric immunoglobulin receptor and Ig alpha-1 chain C region, which are secreted mainly by the transcytotic pathway, were decreased in reflex tears, and mammaglobin-B, clusterin, and cystatin S and SN were decreased in reflex tears [91]. These proteins in tears may be the key players in the protection and maintenance of the dynamic balance of the ocular surface among individuals with DED. Finally, MMP-9 is another quantifiable target to diagnose DED [74,75]. InflammaDry is a point-of-care MMP-9 immunoassay device to qualitatively assess MMP-9 levels in tears [76,77]. One study reported a sensitivity of 85% and specificity of 94% for InflammaDry in diagnosing DED [78].

Proteomics analyses have been applied to study DED associated with systemic diseases including thyroid eye disease, SS, and graft-versus-host disease. Thyroid eye disease (or thyroid-associated ophthalmopathy) is the most prevalent extrathyroidal manifestation of Grave’s disease. DED results from thyroid-associated ophthalmopathy (TAO) owing to lacrimal gland involvement, eyelid retraction, impaired Bell’s phenomenon, reduced blinking, and proptosis [92]. Matheis et al. [93] analyzed the tear-fluid proteome of TAO patients via mass spectrometry. This study shows that patients with TAO displayed an increase in inflammatory proteins (i.e., POTE ankyrin-domain family member 1) and a reduction in protective and anti-inflammatory proteins (i.e., proline-rich lacrimal protein 1 (PROL1), protein-rich protein 4 (PRP4), and annexin A1) and a significantly different protein panel (PRP4, PROL1, and UDP-glucose-dehydrogenase) in individuals with TAO and those with DED and/or healthy controls. Therefore, the spectrum of inflammatory and protective proteins might be a useful indicator for DED activity in patients with TAO.

SS is an autoimmune disorder mostly targeting exocrine glands, especially salivary and lacrimal glands [94]. Li et al. [95] examined and compared the tear proteome of patients with SS, those with DED, and healthy participants. The results showed that DED in SS patients is associated with an altered proteomic profile with dysregulated expression of proteins involved in inflammation, apoptosis, immunity, and oxidative stress. Characterization of these proteins would yield potential diagnostic markers and therapeutic targets.

Graft-versus-host disease (GVHD) is a major cause of mortality and morbidity following allogeneic hematological stem cell transplantation (HSCT). The immune response destroys the conjunctiva and lacrimal gland tissues, resulting in decreases in tear production and DED onset [96]. Cocho et al. [97] reported a significant decrease in tear level epidermal growth factor and the IFN-γ-induced protein-10 (IP-10):CXCL10 ratio, whereas IL-1 receptor-α, the IL-8:CXCL8 ratio and IL-10 levels were significantly elevated as compared with healthy individuals. They suggested a predictive model for diagnosing ocular GVHD based on the IP-10: CXCL10 ratio adjusted to sex and age, finding a sensitivity of 86.36% and a specificity of 95.24% [97]. Jung et al. [98] reported a significant increase in tear IL-2, IL-10, IL-17α, IFN-γ, IL-6, and TNFα levels in ocular GVHD, with adjustment for age, sex, and time after HSCT showing that the diagnostic capabilities of these cytokines were significant and independent. Moreover, IL-10, IL-6, and TNFα displayed the strongest correlation to GVHD severity relative to other cytokines [98].

Remarkably, analyses of ocular GVHD has shown that DED was already present in a significant percentage of patients suffering from hematological diseases before HSCT [99,100]. A comprehensive ophthalmic assessment pre- and post-HSCT is recommended for the early treatment and potential reduction of postoperative ocular damage [101,102].

### 3.3. Bioengineering Approaches

The increasing research expense for developing new drugs [103] and the pronounced difference in drug effects between humans and other species [104] have resulted in emerging techniques to model human physiology in preclinical studies [105]. Organ-on-a-chip technology introduced three-dimensional (3D) techniques to mimic in vivo conditions by employing microfluidics and bioengineering [104]. Human blinking eye-on-a-chip is an example of this technology for ophthalmology [106]. In this model, 3D shell scaffolds are used to generate similar curvatures to corneas, followed by impregnating with primary human keratocytes and sandwiching between a microfluid channel and a circular chamber. The epithelial cells are then placed on the scaffold surface by a color-coded method, with green fluorescence in the center and red fluorescence on the periphery of the scaffold surface. The 3D-printed eyelids, which mimic normal blinking, are electromechanically actuated, thereby allowing recapitulation of tear-film spreading and ocular-surface hydration.

Corneal organoid, the minatory cornea produced by tissue engineering, mimics in vivo conditions to allow the study of organ development, course, and treatment of diseases. Pluripotent stem cells are an alternative source compared to embryonic stem cells for organoid generation [107]. Microcornea or corneal organoids are produced in later stages of retinal organoid formation [108] and can be used for drug screening, disease modeling (e.g., DED), and tissue replacement.

**Figure 4:**
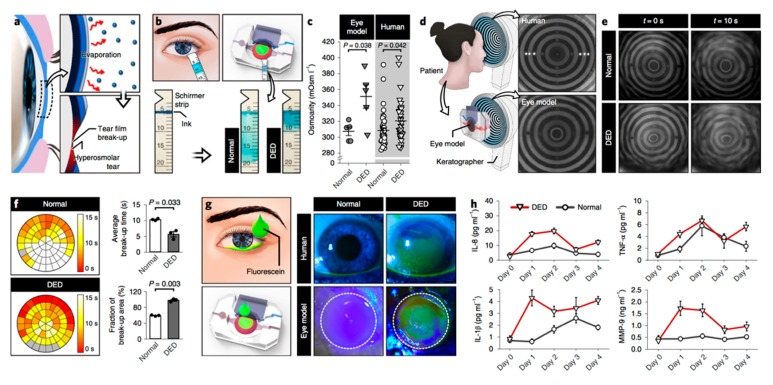
An engineered model of evaporative dry eye disease. (**a**) Evaporation causes the break-up of the tear film and increases tear osmolarity that together leads to a loss of homeostasis. (**b**) Absorption of tears into the Schirmer strips in the healthy and dry eye models. Tear absorption is visualized by the smearing of the blue ink within the strips. (**c**) Tear osmolarity in the DED (closed triangle) and the normal (closed circle) models. Human clinical data of osmolarity are from normal subjects (open circle) and DED subjects (open triangle). (**d**) Keratographs showing concentric rings projected on the human ocular surface (top) and the engineered ocular surface (bottom). (**e**) Representative images of projected ring patterns on the engineered ocular surface of the healthy (top row) and the DED (bottom row) groups captured at *t* = 0 s (left column) and *t* = 10 s (right column). (**f**) Spatial mapping of tear film break-up time in the normal (top) and the DED (bottom) models. Different colors in the representative circular heat maps indicate different tear break-up times. (**g**) Fluorescein staining of the eye model and human subjects. (**h**) Concentrations of inflammatory mediators (IL-8, TNF-α, IL-1β, and MMP-9) in the normal (circle) and the DED (triangle) groups plotted against the duration of culture. Figure taken from Jeongyun Seo et al. with permission [109].

Given that immunometabolic alterations play a principal role in DED pathogenesis, it will be very helpful to include both immune factors and metabolic elements in the engineered models. Jeongyun Seo et al. recently developed an eye-on-a-chip model that can mimic the pathogenesis of DED [109]. This model includes a blinking corneal surface and can be used to induce a DED phenotype (see Figure 4). Jeongyun Seo et al. assessed IL-8, TNF-α, IL-1β, and MMP-9 expression in their model after inducing DED and monitored the response of these cytokines to DED treatment by administering lubricin.

## 4. Emerging DED-Treatment Strategies

DED treatment depends on its severity and the presence of any underlying conditions. Systemic diseases, if any, must be controlled. Improvement of tear quality and quantity with artificial tears, anti-inflammatory medication, diet and lifestyle modification, and treatment of associated eyelid diseases are the primary therapeutic strategies.

Tiny disk-like membranes called nanowafers containing drugs can be applied on the eye for sustained drug release instead of repeated administration of drops. Sustained release of dexamethasone by loaded nanowafers showed an equivalent effect to (either oral or topical) betamethasone administered twice daily and restored ocular-surface smoothness and corneal epithelial-barrier function while reducing levels of inflammatory cytokines [110]. Another study revealed that nanowafer-loaded dexamethasone decreased inflammatory cytokine expression, especially in later stages of inflammation [111]. Additionally, a vehicle-controlled clinical trial showed that treatment with 0.1% fluorometholone preserved corneal integrity under environmental stress [112]. Immunomodulatory drugs modify the function of the immune system to control autoimmune diseases. Rapamycin (sirolimus), an immunomodulatory drug, reduces inflammation in DED. Treatment of non-obese diabetic mice (NOD) with rapamycin twice daily for 12 weeks decreased lymphocyte infiltration into lacrimal gland lysates. Moreover, tear secretion was increased following topical administration of rapamycin, although, GC density did not change, while decreasing cathepsin S levels in the lacrimal glands and tears of the mice [113].

Essential FAs modulate the function of the immune system by conversion into pro-inflammatory and anti-inflammatory cytokines; a previous study reported that ω3 FAs convert into anti-inflammatory cytokines, and ω6 FAs convert into pro-inflammatory cytokines [114]. They showed that consumption of ω3 supplements for 90 days reduced tear osmolality, OSDI, ocular redness, and ocular-surface staining, thereby increasing tear-film stability and that these effects were heightened when using the phospholipid form of ω3 (krill oil) as compared with the triacylglyceride form (fish oil) [115]. Ovariectomy decreases lacrimal production according to Schirmer test results; however, supplementation with n-3 PUFAs, docosahexaenoic acid (DHA), eicosapentaenoic acid (EPA), and α-lipoic acid restored partial lacrimal production while only alkaline phosphatase (ALP) resulted in complete restoration. SOD and aconitase levels were not altered by ovariectomy or supplementation with FAs, although supplementation with ALP increased glutathione peroxidase (GPx) activity. Furthermore, ovariectomy decreased nitrite and nitric oxide levels on the ocular surface, which was not recovered by FAs in the conjunctiva but was restored by ALP in the cornea. Moreover, DHA, EPA, and ALP restored nitrite and nitric oxide levels in lacrimal glands, and EPA and DHA also increased MDA levels in lacrimal glands. DHA, EPA, and ALP function by avoiding microvilli loss, preventing cellular-junction irregularity, and preventing ovariectomy-induced cellular desquamation [116]. Another study showed that oral omega-3 essential FA supplementation for symptomatic DED in computer users improved TBUT and results from the Schirmer test while also changing the cytology of GCs and epithelial cells [117]. Administration of topical 0.2% omega-3 FA mixed with hyaluronic acid reduces the severity of corneal irregularity, which was significantly improved relative to results using hyaluronic acid alone or mixed with 0.02% omega-3 FA [118]. Additionally, adding mineral oil to eye drops significantly increases lipid-layer thickness at the ocular surface of patients with Meibomian gland dysfunction (MGD) and preserves the tear film from evaporation [119].

Eyelid-warming liquefies Meibomian gland secretions and facilitates their release onto the tear film. Eye-mask and eye-bag compresses are devices used to warm the eyelid. Outer and inner eyelid temperatures were significantly increased using an eye-bag compress, and although there was no significant difference in lipid-layer grade and non-invasive TBUT with the eye-mask compress, there was improvement between treatments. Subjectively, the majority of subjects preferred eye-bag compresses over eye-mask compresses [120].

In a survey comparing the effects of TNFα–stimulated gene/protein-6 (TSG-6), topical prednisolone, and topical cyclosporine (CsA) on reducing DED-associated changes in NOD mice, all three increased tear production and conjunctival GC count. Additionally, 1% prednisolone drops did not decrease corneal epithelial staining, whereas TSG-6 and CsA (0.05%) did. Moreover, topical administration of TSG-6, Restasis (CsA), and Pred Forte (prednisolone acetate, 1%) significantly decreased transcript levels of *Tnfa* and *Ifng* at the ocular surface and intraorbital glands, and Pred Forte also increased epithelial-cell apoptosis and decreased corneal thickness [121]. In rat models, diclofenac, a non-steroidal anti-inflammatory drug, prevented DED changes without decreasing tear-fluid volume and reduced cell damage and apoptosis induced by hyperosmolarity [122].

CsA is an anti-inflammatory medication used to treat DED. Because of several adverse effects associated with systemic administration of CsA, topical drops are the route of choice for treatment of the ocular surface; however, formulating a safe delivery system for this hydrophobic drug is challenging. Available topical CsA brands are often associated with adverse effects, including ocular burning, foreign-body sensation, and epiphora [123]. However, 6- and 12-week treatment with topical CsA 0.05% ophthalmic emulsion twice daily increased the conjunctival density of GCs and transforming growth factor β2–positive GCs, suggesting that this treatment increases the production of the immunoregulatory factor TGF-β2 by increasing conjunctival GCs [124]. Another study showed that vitamin B12 supplementation restored tear volume and TBUT in a murine model of DED, with 1-month treatment with B12 and 0.15% hyaluronic acid decreasing oxidative stress and OSDI [125].

Oxidative stress is involved in DED pathogenesis. A previous study assessed the anti-oxidative effect of SkQ1, a synthetic antioxidant, on preventing general-anesthesia-induced DED, showing that premedication with instilled SkQ1 (7.5 μM) displayed preventive effects against pathological corneal changes after recovery and completely neutralized clinical signs of DED as early as the first day of the post-anesthetic period. Treatment after anesthesia was 1 week, with these findings suggesting that SkQ1 protected the corneal epithelium rather than participated in corneal wound healing. Moreover, SkQ1 administration increased GPx and glutathione reductase activities, accelerated normalization of SOD and other oxidant levels in tear fluid, increased IL-10 secretion, accelerated the recovery of IL-4 levels, and suppressed TNFα and IL-6 secretion. Furthermore, MDA concentration decreases significantly in animals premedicated with SkQ1 before anesthesia relative to control animals, supporting its anti-oxidative effect [126].

Diquafosol tetrasodium (DQS) is a purinergic P2Y_2_ agonist that reportedly improves DED via several mechanisms of action, including improvement of fluid transport, secretion of mucin from the conjunctival epithelium, and stimulation of lipid production [127]. Ikeda et al. [128] investigated the effect of 3% DQS eye drops on functional changes of MGD in *Sod1*^−/−^ mice, observing that DQS instillation increased aqueous tear production. Additionally, *Sod1*^−/−^ mice displayed significantly lower TBUT, and a 2-week treatment significantly decreased corneal fluorescein staining and lissamine green staining. Moreover, they found increased *cytokeratin-4* and IL-*13* expression in Meibomian gland acinar epithelium and decreased transglutaminase-1 mRNA and protein levels in DQS-treated mice [128].

Thrombospondin-1 (TSP-1) activates TGF-β, which plays an immunomodulatory role at the ocular surface [129]. Tan et al. [130] found that TSP-1 levels are upregulated in the corneas of mice with DED, and interestingly, expression of MHC II by bone-marrow-derived DCs (BMDCs) decreased significantly following co-culture with epithelial cells from DED mice. Further addition of recombinant (r)TSP-1 potentiated the suppressive effect of epithelial cells on BMDC maturation, whereas TSP-1 blockade ameliorated this effect. Furthermore, rTSP-1 decreased the Th17 population in draining lymph nodes of mice with DED and decreased cytokine expression in the conjunctiva and cornea relative to levels in controls. Studies suggest that the interaction between pro-inflammatory and immune-regulatory Th17 cells play a principal role in DED pathogenesis [20,32]. Sustained release of CCL22 from microspheres injected locally in lacrimal glands prevented the loss of tear production and GCs, increased CD4+ T cells in regional draining lymph nodes, increased CD4+ IFN-γ+ T cells, and decreased CD4+ Foxp3+ T cells in lacrimal glands and corneas in DED animal models [131].

In summary, most of the available treatment strategies are focused on increasing ocular surface humidity and decreasing its osmolarity and inflammation. Future medications and supplementations improve oxidative condition and metabolic regulation at the ocular surface. This strategy should be considered in future studies and clinical trials. For example, TLR signaling pathway is one of the presumed pathways connecting oxidative stress and inflammation and is the target of some proposed supplementations in the recent studies which can be assessed in future DED treatment development studies [132,133].

## 5. Future Directions

DED poses a significant clinical challenge. Previous reports reveal discrepancies between dry eye signs and symptoms [134,135], indicating a need for new disease markers with higher predictive values. Identification of such markers often requires the use of new measurement techniques [80]. In this review, we highlighted recent advances in analytical chemistry, microbiology, and bioengineering and their applications in DED diagnosis and treatment. In particular, we highlighted the newly identified immunometabolic pathways and microbiota-related factors that are involved in DED pathogenesis. The newly found biomarkers offer hope for improved diagnosis and disease prediction; however, further studies are required to determine the most predictive biomarkers regarding disease severity. Modeling approaches, including machine-learning techniques, and artificial intelligence, are being increasingly utilized by ophthalmologists, and can be used to identify clinically meaningful patterns in the data. Additionally, in vitro disease models, such as eye-on-a-chip, will provide efficient screening platforms for future drug development.
